# Multidisciplinary rehabilitation network enhances outcomes after nerve transfer in brachial plexus birth injury

**DOI:** 10.1007/s00381-026-07216-w

**Published:** 2026-03-17

**Authors:** Alejandro J. Friedman, Victoria Robbins, Megan Gotlieb-Horowitz, Mandana Behbahani, Susan Durham, Erin Meisel, Steven M. Koehler

**Affiliations:** 1 Division of Hand Surgery, Department of Orthopaedic Surgery, Montefiore Einstein, 1250 Waters Place, Tower One, 11 Floor, Bronx, NY 10461 USA; 2 Division of Hand Therapy, Motion PT Group, Bronx, NY 10461 USA; 3 Department of Neurological Surgery, Montefiore Einstein, Bronx, NY 10461 USA; 4 Division of Pediatric Neurosurgery, Children’s Hospital Los Angeles, Los Angeles, CA USA; 5 Department of Orthopaedic Surgery, Children’s Orthopaedic Center, Children’s Hospital Los Angeles, Los Angeles, CA USA

**Keywords:** Nerve transfer, Brachial plexus birth injury, Rehabilitation, Outcomes, Spinal accessory to suprascapular nerve transfer, External rotation

## Abstract

**Purpose:**

Outcomes after brachial plexus birth injury (BPBI) vary widely, highlighting the need for rehabilitation strategies that reliably enhance functional recovery. We hypothesized that a coordinated, interdisciplinary rehabilitation network involving surgeons and occupational therapists improves functional outcomes after spinal accessory to suprascapular nerve (SAN-SSN) transfer. We compared outcomes from two academic centers with similar surgical indications and techniques but distinct rehabilitation models.

**Methods:**

We reviewed 25 infants who underwent SAN-SSN transfer from 2022 to 2024. Institution A used a coordinated multidisciplinary rehabilitation model in which the surgeon partnered directly with specialized therapists. Patients at institution B self-selected therapy sites. Pre- and postoperative external rotation Active Movement Scale (AMS) scores were collected and analyzed.

**Results:**

Both cohorts showed significant improvement in external rotation (ER) (*P* < 0.05). Median postoperative AMS scores were higher at institution A (7; interquartile range [IQR] 5.5–7) than at institution B (4; IQR 3–5). Functional recovery (AMS ≥ 6) occurred in 72.7% of institution A patients versus 14.3% at institution B (*P* = 0.005). Within institution B, on-site therapy produced significantly better outcomes than off-site therapy. Patients at institution A had 11.8-fold greater odds of achieving higher AMS scores than patients at institution B (*P* = 0.018); these differences were not significant when comparing institution A to institution B’s on-site subgroup.

**Conclusions:**

Surgeon-directed, expert-guided therapy is associated with superior recovery after SAN-SSN transfer for BPBI. These findings support the impact of a scalable model which prioritizes timely, consistent, and coordinated postoperative rehabilitation.

**Level of Evidence:**

III, retrospective cohort comparison.

## Introduction

Brachial plexus birth injury (BPBI) remains a significant cause of upper limb impairment in children, and persistent deficits may lead to lifelong disability [14, 30, 33]. Although many infants recover spontaneously, a substantial proportion develop limitations that impair activity, cause deformity, and reduce quality of life [15, 22, 39, 42]. Early identification and timely reconstruction are crucial; failure to meet established recovery benchmarks, such as the “Cookie Test” at nine months, typically prompts surgical intervention [4, 39, 41, 45]. Without intervention, children face high risks of poor muscle recovery and progressive secondary sequelae such as internal rotation contracture and glenohumeral dysplasia (GHD) [20, 24].

Nerve reconstruction for non-recovering injuries can be achieved through autologous grafting or distal nerve transfers [35]. The spinal accessory nerve to suprascapular nerve (SAN-SSN) transfer is widely used but yields variable results [7, 23, 29, 31, 36, 38, 46]. Prior studies attribute this variability to factors such as passive motion limitations and injury severity [3, 21, 36]. However, our clinical experience suggests that consistent access to expert, highly coordinated therapy plays a more decisive role. Nerve transfers require specialized rehabilitation to activate donor nerves, retrain reinnervated muscle, and reinforce emerging motor patterns [18, 19]. Effective recovery depends on reliable communication among surgeons, therapists, and caregivers [17–19, 39].

Socioeconomic and geographic barriers frequently impede access to specialized therapy [10, 12]. We hypothesized that a structured, surgeon-coordinated multidisciplinary rehabilitation network improves functional outcomes after nerve transfer. To test this, we examined outcomes following SAN-SSN transfer in infants managed at two academic centers with similar surgical practices but different therapy models.

## Methods

This study received Institutional Review Board approval and adhered to the ethical standards established in the 1964 Declaration of Helsinki.

From 2022 to 2024, two surgeons at separate institutions performed 29 SAN-SSN transfers in infants with non-recovering upper trunk (C5-6) or extended upper trunk (C5-7) BPBI. Institution A treated 15 patients; institution B treated 14. Both surgeons used the same operative technique, including internal rotation contracture releases when needed to restore full passive motion—all patients began their recovery with baseline full passive range of motion.

### Institution A

The surgeon partnered directly with an extensive network of specialized pediatric hand therapists located on-site and in vetted satellite clinics. The surgeon and senior therapists, who specialize in BPBI, jointly selected a therapy site based on geographic convenience, scheduled the initial appointment, and maintained continuous communication. Therapists followed a standardized protocol with real-time senior plexus therapist oversight to ensure consistent, coordinated management.

### Institution B

Institution B used a more decentralized model: families independently selected therapy sites. Some chose on-site therapy when able; others attended unaffiliated external clinics. Institution B had no structured communication with external therapists.

Rehabilitation protocols were nominally identical across institutions; the primary distinction was the presence or absence of coordinated surgeon–therapist communication.

The primary outcome was the Active Movement Scale (AMS) scores for shoulder external rotation (ER), obtained pre- and postoperatively. We selected AMS as our outcome measure due to its ability to minimize inter-observer variability in multicenter studies and its strong reliability in reflecting active shoulder strength [1, 11, 36]. ER was selected to decrease confounding and for its importance: ER is essential for functional independence in daily activities [9, 26, 32, 33, 43, 46]. While the SAN-SSN transfer reinnervates the supraspinatus to aid shoulder abduction—contributing up to 90° of motion—it does not directly reinnervate the shoulder flexors [33, 36]. Endpoints included functional recovery, defined a priori as AMS ≥ 6, plateau of recovery by 12 months, or need for conversion to additional surgical procedures [5, 6, 8].

We analyzed demographic data with descriptive statistics. Wilcoxon signed-rank tests compared median AMS changes within groups; Fisher’s exact or chi-square tests compared categorical variables. Proportional odds ordinal regression models evaluated postoperative AMS scores while adjusting for preoperative AMS scores. Statistical significance was set at p < 0.05. Analyses were performed using R software version 4.2.3.

## Results

We excluded four institution A patients who underwent simultaneous tendon transfer, leaving 11 patients at institution A and 14 at institution B. Baseline characteristics and preoperative AMS scores were similar (Table 1).

**Table 1:** Demographics and baseline active movement scale scores for institutions A and B

Variable	Institution A (*n* = 11)	Institution B (*n* = 14)	Statistical test	*P*-value
	Mean (SD)	Mean (SD)		
Sex	6 male, 5 female	6 male, 8 female	Chi-square test	0.860
Age at surgery (mo)	5.64 (3.76)	8.82 (2.55)	Independent *t*-test	**0.028**
Follow-up duration (mo)	14.86 (6.01)	20.97 (5.56)	Independent *t*-test	**0.017**
Baseline ER AMS	2 (2.5–4.75)	1 (0–1)	Wilcoxon signed rank	0.138

**Fig. 1:**
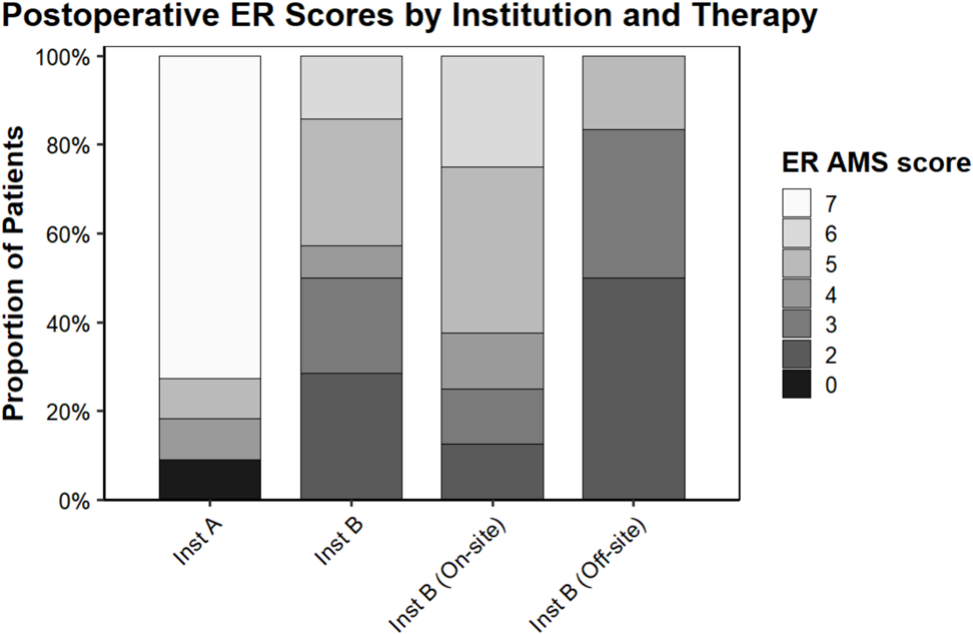
Stacked bar graph representing postoperative external rotation Active Movement Scale score of institution A and institution B, as well as the on-site therapy and off-site therapy subgroups of institution B patients

Both institutions showed significant postoperative improvement (*P* < 0.05) (Table 2; Fig. 1). At institution A, 72.7% of patients reached AMS ≥ 6. Only 14.3% of institution B patients achieved this level (*P* = 0.005). Additionally, 90.9% of institution A patients achieved AMS ≥ 4 versus 50% of institution B patients (*P* = 0.042) (Table 3).

**Table 2:** Comparison of pre and postoperative external rotation active movement scale scores by Wilcoxon signed rank test

Hospital	Preoperative	Postoperative	*W*	*P*-value
	Median (IQR)	Median (IQR)		
**Institution A**	2 (2.5–4.75)	7 (5.5–7)	*W* = 36	**0.013**
**Institution B**	1 (0–1)	4 (3–5)	*W* = 78	**0.002**
*On-site therapy*	1 (0–2)	5 (3.75–5.25)	*W* = 28	**0.022**
*Off-site therapy*	1 (0–1)	3 (2–3)	*W* = 15	0.055

**Table 3:** Proportional odds ordinal regression models of pairwise cohorts. Models are adjusted for pre-op ER AMS

	Odds ratio	95% CI	*P*-value
**Institution**			
* A*	11.8	1.73–113.0	**0.018**
* B*	Ref	-	**-**
**Institution**			
* A*	7.83	1.10–73.6	0.053
* B (on-site only)*	Ref	-	**-**
**Institution B**			
* On-site therapy*	4.87	0.51–62.7	0.186
* Off-site therapy*	Ref	-	**-**

Outcomes at institution B varied by therapy location. Patients who received on-site therapy showed significant improvement postoperatively; those treated off-site did not. Ordinal regression demonstrated that institution A patients had 11.8-fold greater odds of achieving higher AMS scores than institution B overall (Table 3). When limited to institution B’s on-site subgroup, differences were no longer significant, indicating that therapy coordination—not institutional resources—drove the observed benefit (Table 2 and Fig. 1).

Functional recovery was defined as AMS ≥ 6 [5, 6, 8]. The rate of functional recovery was significantly higher at institution A (72.7%) compared to institution B (14.3%) (*P* = 0.005). Similarly, 90.9% of institution A patients versus 50% of institution B patients achieved AMS ≥ 4 (*P* = 0.042) (Table 4).

**Table 4:** Comparison of functional recovery of external rotation between institutions A and B

Outcome	Institution A (n, %)	Institution B (n, %)	Fisher’s *P*-value
External rotation ≥ 6	8 (72.7%)	2 (14.3%)	**0.005**
External rotation ≥ 5	9 (81.8%)	6 (42.9%)	0.099
External rotation ≥ 4	10 (90.9%)	7 (50.0%)	**0.042**

## Discussion

This study demonstrates that the rehabilitation model strongly influences recovery after SAN-SSN transfer. Although both institutions demonstrated meaningful postoperative improvement using identical operative techniques and postoperative protocols, coordinated surgeon–therapist communication at institution A produced markedly superior functional outcomes.

The literature consistently reports wide variability in recovery after SAN-SSN transfer, with unsatisfactory ER outcomes occurring in 59–80% of patients and high rates of secondary procedures [23, 32, 36]. For example, Manske et al. reported that only 24% of their patients achieved an ER AMS score of 5 or greater, although these results still outperformed outcomes in patients treated with nerve grafts [23]. Furthermore, 40% of their cohort required additional surgery. Seruya et al. reported a mean ER AMS score of 2 at two years postoperatively in 74 patients, 46 of whom received nerve transfers, while O’Grady et al. reported a mean ER AMS score of 4.3 in 14 patients at a similar time point [31, 37]. Our cohort showed a notably low reoperation rate, with only one patient requiring additional surgery. This patient was a 16-month-old global plexus who underwent late SAN-SSN transfer after failure of primary grafting 12 months earlier, to result in ER (AMS 0).

The patients at institution A were statistically significantly younger at the time of surgery than those at institution B, reflective of the closed-loop surveillance system at institution A, which ensures early referral for any neonate with abnormal upper-extremity exam findings. Although this age difference reached statistical significance, it does not represent a clinically meaningful disparity. Functional outcomes after nerve transfer remain stable until approximately 12 months of age, after which results begin to attenuate [39]. All patients in our study underwent SAN–SSN transfer well before this window. Moreover, comparisons of nerve transfer patient outcomes between the 6-month and 9-month surgical timepoints, which are essentially the mean ages of the two cohorts, have failed to show superiority of earlier or later surgery [2,. Other recent literature reports excellent outcomes even in considerably older children, further reinforcing that the modest age variation between cohorts is unlikely to explain the substantial differences observed in postoperative recovery [13, 40]. Instead, this difference should be viewed as an additional advantage of institution A’s coordinated model, which integrates early identification, automatic referral pathways, and structured communication among caregivers, surgeons, and highly trained therapists.

These findings highlight the essential role of expert, consistent, and accessible therapy in nerve transfer recovery. Given the socioeconomic and geographic disparities that often limit access to specialized care, models like institution A’s can help mitigate inequities and increase patient access to expert care [10, 12, 19, 28]. Prior work comparing these cohorts demonstrated similar Area Deprivation Index scores but significantly lower Child Opportunity Index at institution A, reinforcing the added value of a coordinated therapy network that supports families regardless of geographic or socioeconomic constraints [44].

Limitations include the retrospective design, small sample size, lack of blinding, and potential selection and observer bias. Although both surgeons used the extended anterior approach, variations in surgical technique across other centers may influence the translation of our reported outcomes [3] [16, 25]. Although factors such as age at surgery, injury severity, and internal rotation contracture influence recovery, the most meaningful predictor in this study was the postoperative therapy model [3, 34, 39].

A coordinated rehabilitation network grounded in close surgeon–therapist collaboration significantly improves functional outcomes following SAN-SSN transfer for BPBI. Implementing similar models across institutions may enhance access, reduce disparities, and improve recovery for infants with BPBI.

## Data Availability

The data that support the findings of this study are not openly available to protect patient privacy but may be made available from the corresponding author upon reasonable request.
